# Optimizing Within-Subject Experimental Designs for jICA of Multi-Channel ERP and fMRI

**DOI:** 10.3389/fnins.2018.00013

**Published:** 2018-01-23

**Authors:** Jain Mangalathu-Arumana, Einat Liebenthal, Scott A. Beardsley

**Affiliations:** ^1^Department of Biomedical Engineering, Medical College of Wisconsin, Marquette University, Milwaukee, WI, United States; ^2^Department of Neurology, Medical College of Wisconsin, Milwaukee, WI, United States; ^3^Department of Psychiatry, Brigham and Women's Hospital, Boston, MA, United States; ^4^Clinical Translational Science Institute, Medical College of Wisconsin, Milwaukee, WI, United States

**Keywords:** fMRI, ERP, EEG, independent component analysis (ICA), multimodal neuroimaging, modeling, simulation, data fusion

## Abstract

Joint independent component analysis (jICA) can be applied within subject for fusion of multi-channel event-related potentials (ERP) and functional magnetic resonance imaging (fMRI), to measure brain function at high spatiotemporal resolution (Mangalathu-Arumana et al., 2012). However, the impact of experimental design choices on jICA performance has not been systematically studied. Here, the sensitivity of jICA for recovering neural sources in individual data was evaluated as a function of imaging SNR, number of independent representations of the ERP/fMRI data, relationship between instantiations of the joint ERP/fMRI activity (linear, non-linear, uncoupled), and type of sources (varying parametrically and non-parametrically across representations of the data), using computer simulations. Neural sources were simulated with spatiotemporal and noise attributes derived from experimental data. The best performance, maximizing both cross-modal data fusion and the separation of brain sources, occurred with a moderate number of representations of the ERP/fMRI data (10–30), as in a mixed block/event related experimental design. Importantly, the type of relationship between instantiations of the ERP/fMRI activity, whether linear, non-linear or uncoupled, did not in itself impact jICA performance, and was accurately recovered in the common profiles (i.e., mixing coefficients). Thus, jICA provides an unbiased way to characterize the relationship between ERP and fMRI activity across brain regions, in individual data, rendering it potentially useful for characterizing pathological conditions in which neurovascular coupling is adversely affected.

## Introduction

Electrophysiological and hemodynamic measures of brain function vary in terms of their spatial and temporal resolution and the relation of the measured signals to the underlying neural activity (direct vs. indirect, respectively). Electroencephalography (EEG) measures brain function on a millisecond temporal scale and centimeter spatial scale in the form of electrical fields generated on the scalp by the synchronous activity of large populations of neurons. Functional magnetic resonance imaging (fMRI) of the blood oxygen level dependent response (BOLD) measures neural activity indirectly via variations in blood oxygenation that result from changes in the metabolism of electrically active neurons; for a review see (Logothetis, 2008). FMRI measures brain function on a millimeter spatial scale and temporal scale of seconds in the form of slow hemodynamic responses in clusters of neighboring neurons. The complementary spatial and temporal scales of EEG and fMRI, and the possibility of acquiring the activity simultaneously, has been leveraged to examine brain function at a combined millisecond temporal and millimeter spatial scale (Bonmassar et al., 2001; Dale and Halgren, 2001; Horovitz et al., 2002; Liebenthal et al., 2003, 2010; Mulert et al., 2004; Debener et al., 2005, 2006; Bénar et al., 2007; Liu and He, 2008; Liu et al., 2010; Bridwell et al., 2013; Cottereau et al., 2015; Nguyen et al., 2016). Nevertheless, given the differences in the nature of the activity in each modality, an outstanding question is the degree to which they reflect the same neural activity.

Symmetric data fusion techniques provide a mathematical framework to optimize the integration of multimodal neuroimaging data such as combined EEG/fMRI (Rosa et al., 2011; Huster et al., 2012; Uludag and Roebroeck, 2014; Adali et al., 2015a; Cottereau et al., 2015). *Model-driven* approaches rely on *a priori* assumptions of the relationship between the activity in each imaging modality in order to fit a common generative model to the multimodal data (Daunizeau et al., 2007; Luessi et al., 2011; Rosa et al., 2011; Woolrich and Stephan, 2013; Nguyen et al., 2014; Uludag and Roebroeck, 2014; Turner et al., 2016). By explicitly modeling a generative (neural) source for the observed activity, model-driven approaches enable hypothesis-driven analyses of EEG/fMRI data. However, they are necessarily limited by the extent to which the neural sources and the physiological and physical relationships between neuroimaging measures are known.

*Data-driven* approaches, such as those based on blind source separation, attempt to minimize assumptions about the relationship between neuroimaging measures (Correa et al., 2010; Mantini et al., 2010; Sui et al., 2012; Brown et al., 2013; Adali et al., 2015b). Independent component analysis (ICA) uses a linear mixing model to identify statistical relationships between neuroimaging activity when detailed *a priori* models are not available (Calhoun et al., 2006). Such approaches allow neuroimaging datasets from different modalities to interact on an equal footing. Joint ICA (jICA) has been used to identify co-variations between EEG event related potentials (ERPs) and fMRI data across a group of subjects (Group-ICA) (Moosmann et al., 2008; Calhoun et al., 2009; Doñamayor et al., 2012; Edwards et al., 2012; Mijović et al., 2012; Adali et al., 2015a, for review see Sui et al., 2012). We have previously used jICA to identify co-variations between ERP and fMRI activity across experimental conditions within-subject (Mangalathu-Arumana et al., 2012).

In our previous study using an auditory oddball task with four parametrically varying experimental levels (specifically, four types of deviants detectable at 65, 75, 85, and 95% accuracy) in each subject, a single jICA-fMRI component was found to carry all the ERP activity associated with the task (Mangalathu-Arumana et al., 2012). The joint component consisted of temporal, parietal and frontal cortical areas activated at time windows corresponding to the N100 and the P300 ERPs. From a purely analytic standpoint, this finding reflects a similar (i.e., non-separable) dependence on experimental level of all the spatiotemporal elements in the joint component. However, from a physiological standpoint at least two different interpretations should be considered: (1) A tight functional association between brain areas resulted in an entire network effectively functioning in synchrony as a single neural source. (2) Functionally distinct neural sources were not separated into independent components, possibly due to low variability in the data resulting from a small number of independent representations of the ERP/fMRI activity. Specifically, activity was averaged across trials for each of experimental condition, resulting in four separate instantiations of ERP/fMRI activity in the analysis. To distinguish between these interpretations, task-related variability in the data can be increased by increasing the number of independent representations of the ERP/fMRI data. This can be achieved by analyzing single trials, or multiple averages over small numbers of trials in each experimental level. However, for analyses within subject, to increase the number of representations typically requires reducing the signal-to-noise ratio (SNR) of each representation (due to constraints on the total number of trials that can practically be collected within a fixed time frame).

In data-driven analyses, dimension reduction, vis-a-vis model order selection techniques (Stoica and Selen, 2004), is often used to estimate the number of latent sources to avoid overfitting the data. The process of dimension reduction also acts to increase SNR by “filtering” irrelevant signals (i.e., noise). Eigenvalue-based techniques for model order selection that combine principal component analysis (PCA) with information theoretic criteria (e.g., Akaike's information criterion—AIC, and minimum descriptor length—MDL) have been incorporated into ICA toolboxes. However, most model order selection techniques assume that the underlying latent sources are Gaussian distributed and the samples (e.g., fMRI voxels, EEG temporal activity) are independent and identically distributed (Wax and Kailath, 1985). FMRI and EEG activity often violates these assumptions which can lead to inaccurate estimates of model order (Majeed and Avison, 2014). Understanding how inaccurate model-order estimates impact data-driven multimodal neuroimaging analyses (such as jICA) is important for properly interpreting the recovered sources of brain activity.

An objective of the present simulations was to determine the range of variability in ERP and fMRI activity and the noise levels under which jICA can successfully retrieve the spatiotemporal attributes of independent neural sources, within a framework wherein the accuracy of the model order selection is not known precisely. Ideally, ERP and fMRI activity generated by the same neural source would be retrieved in the same joint component; and activity generated by neural sources that do not covary in space and/or time would be retrieved in different components. The impact of neuroimaging variability and noise level on jICA performance was examined in the context of both parametrically varying sources (i.e., systematically varying across representations of the ERP/fMRI data, effectively forming a single functional network), and non-parametrically varying sources (i.e., varying independently across representations of the data, effectively forming independent functional networks).

A second objective of the study was to test the performance of jICA when the relationship between ERP and fMRI measures of brain activity is linear and non-linear. Changes in mass electrical activity of neurons (measured with ERPs) can produce linear or non-linear changes in the hemodynamic response (measured with fMRI), depending on the brain area activated and the rate and intensity of stimulation. At short inter-stimulus intervals (below 4 s), the fMRI response can increase non-linearly relative to the ERP response, especially in cortical association areas. At longer intervals and in cortical sensory areas, the relationship is typically linear (Rees et al., 1997; Mechelli et al., 2000; Birn and Bandettini, 2005; Liu et al., 2010). Brief periods of synchronous activity in a small neuronal assembly may generate a negligible change in local metabolic consumption, and thus may be captured with ERP but not with the much slower fMRI BOLD response (Babiloni and Cincotti, 2004). Conversely, neural activity of limited spatial extent may be visible with BOLD, but located too deep in the brain or electrically oriented such that it does not elicit a significant ERP (Nunez and Silberstein, 2000). Pathological conditions can also lead to highly non-linear (essentially uncoupled) relationships between neural and hemodynamic measures of brain activity (Girouard and Iadecola, 2006). While the performance of jICA with linearly varying signals is relatively well established (Calhoun et al., 2006; Mangalathu-Arumana et al., 2012), to our knowledge its application to non-linear relationships has not systematically been examined.

In order to assess the effects of experimental design and data quality on jICA performance, simulated fMRI/EEG datasets representing the activity of three neural sources with distinct spatiotemporal profiles were constructed using parameters and constraints obtained experimentally from individual data (Mangalathu-Arumana et al., 2012). The simulations show that the ability of jICA to accurately retrieve and separate independent neural sources improves with neuroimaging SNR and number of independent representations of the ERP/fMRI data, and deteriorates when the neuroimaging activity is correlated (i.e., vary parametrically) across representations. JICA performance does not, however, depend on the type of relationship (linear, non-linear, uncoupled) between instantiations of ERP/fMRI activity. The simulations also demonstrate how the type of relationship between ERP and fMRI activity can be retrieved using the common mixtures profile across linked ERP/fMRI jICA components.

## Materials and methods

### Overview of computational model and simulations

**Figure 1:**
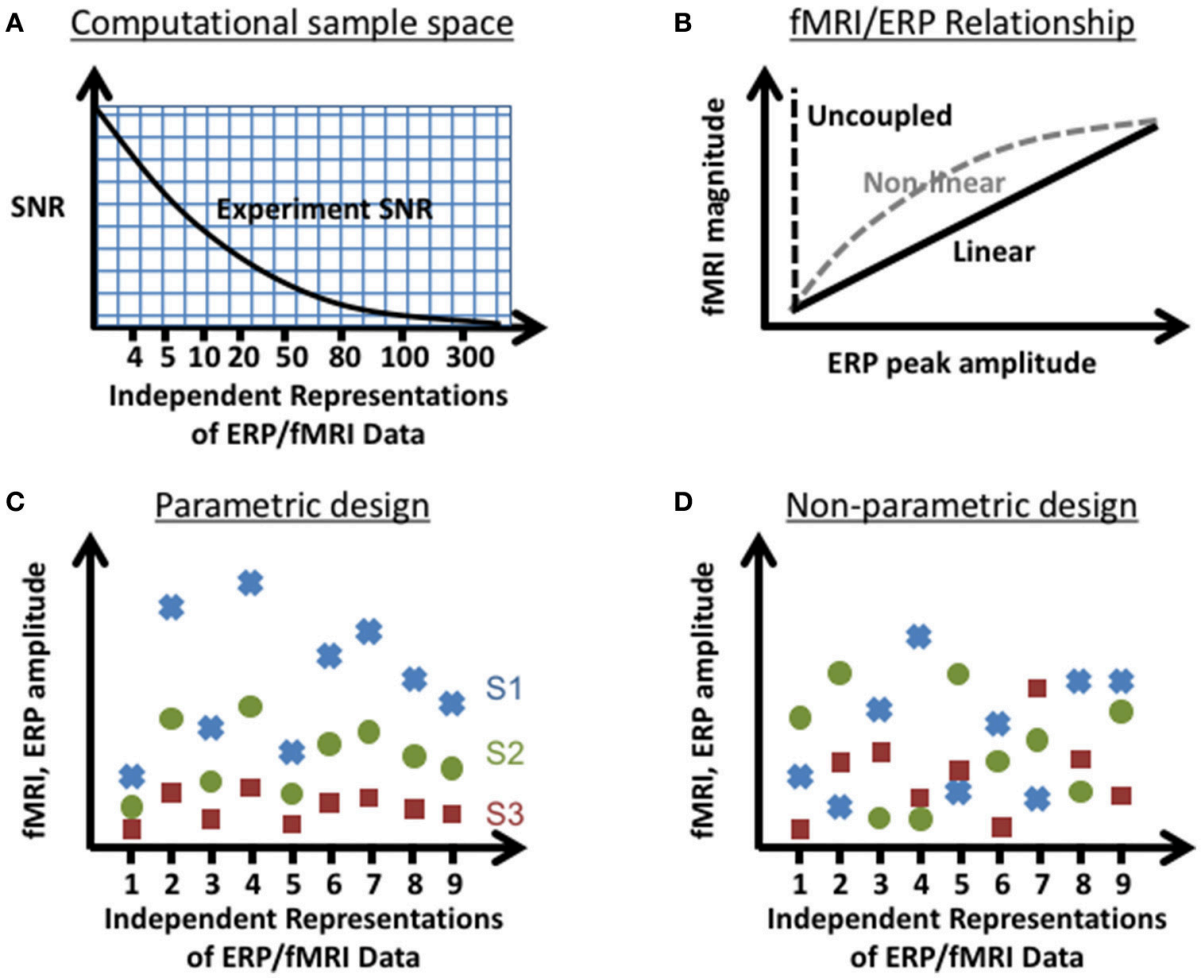
Experimental parameters tested in the computational simulations. Illustration of the parameter space tested: **(A)** SNR vs. number of independent representations of the data (e.g., trials, trial averages of joint ERP/fMRI activity etc.), **(B)** linear and non-linear relationships between instantiations of ERP and fMRI activity, and sources that vary **(C)** parametrically and **(D)** non-parametrically (shown here for a linear relationship between fMRI and ERP) within an experimental design. In the parametric experimental design, the change in measured ERP/fMRI amplitudes of separate neural sources (S1–S3; red square, green circle, blue x respectively) varied systematically across representations of the data. In the non-parametric experimental design, the changes in measured ERP/fMRI amplitudes were different for each source across representations of the data.

**Figure 2:**
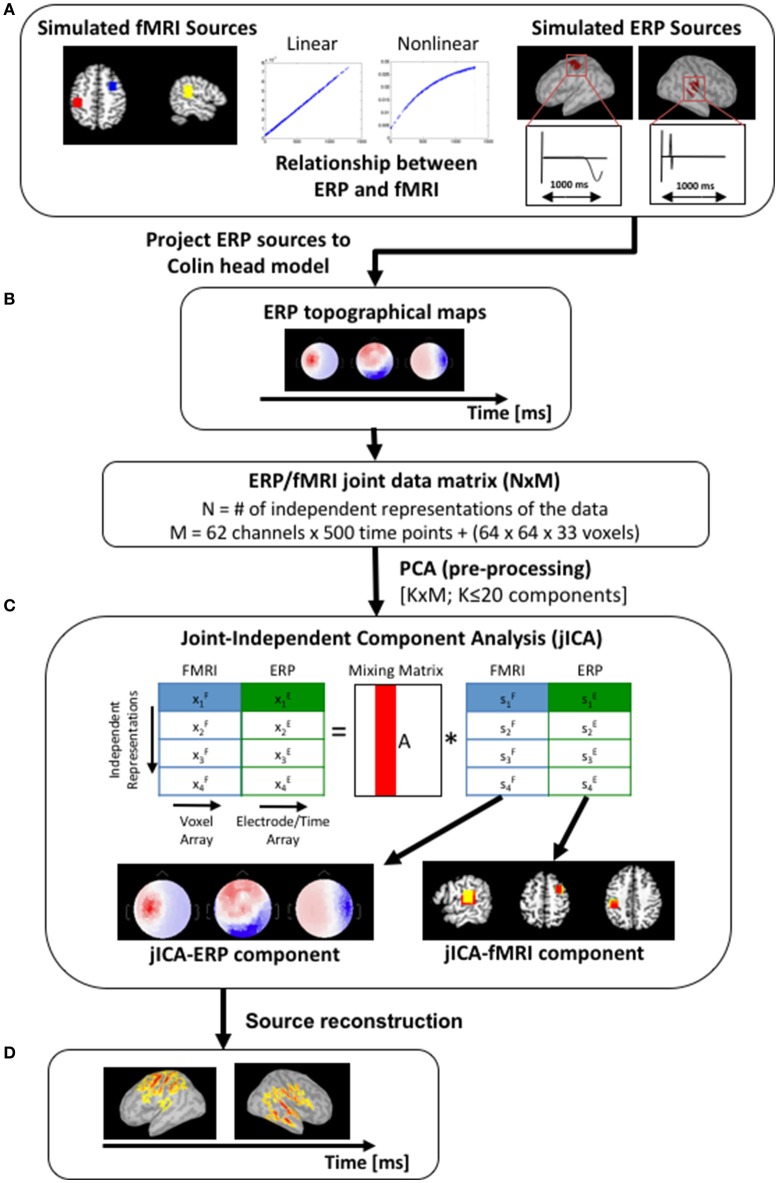
Simulation work-flow. **(A)** Three sources were simulated, with fMRI activity in the right prefrontal cortex, right temporoparietal cortex, and left motor cortex (left panel—blue, yellow and red squares respectively). Volumetric fMRI data corresponding to the right temporoparietal and left motor sources were projected onto a cortical surface mesh and simulated with gaussian-weighted sinusoidal temporal profiles (right panel). The fMRI and ERP activity of the temporoparietal and motor sources co-varied linearly (simulation #1) or non-linearly [f(x) = 1−exp(x), simulation #2], and the prefrontal source was uncoupled [f(x) = 0]. **(B)** ERP topographical maps (shown here for a single source at three time points) were generated by forward projecting the temporoparietal and motor sources onto the scalp partition of the Colin head model. **(C)** The simulated fMRI and ERP datasets were initially concatenated into a single row to create a joint-matrix across imaging modalities and independent representations of the data (Mangalathu-Arumana et al., 2012). PCA was applied to the joint-matrix to whiten the signal and to limit the dimensionality of the dataset to twenty components. JICA was then performed on the PCA components. The resulting jICA components consisted of a spatial jICA-fMRI map and a spatiotemporal jICA-ERP map. **(D)** Source maps of the jICA-ERP channel data were computed and projected onto the Colin cortical surface model to facilitate comparison with the jICA-fMRI maps.

In order to characterize the trade-offs between choices in experimental design and jICA performance, the ability of jICA to recover brain activity measured with fMRI and ERP in space and time was examined for parametrically and non-parametrically varying sources along three dimensions (summarized in Figure 1); the number of independent representations of the ERP/fMRI data (e.g., trial or trial-averaged instantiations of ERP/fMRI activity), the SNR of fMRI and ERP data, and the nature of the relationship between the fMRI and ERP activity (linear, non-linear, uncoupled). Multi-channel ERP waveforms and whole-brain fMRI maps were simulated for three sources of brain activity in regions previously implicated in auditory oddball detection (Liebenthal et al., 2003; Mangalathu-Arumana et al., 2012), in the right prefrontal cortex, right temporoparietal cortex, and left motor cortex (Figure 2—top left; blue, yellow, and red squares, respectively).

Analyses were performed using a 1,024-core high-performance computing cluster (8 cores/node, 24 GB RAM per node). Simulations were implemented on the cluster as a series of distributed processes, where each combination of SNR and the number of representations of the ERP/fMRI data was run as an independent simulation on a single node. FMRI statistical maps were computed in AFNI (https://afni.nimh.nih.gov/) and loaded into Matlab (Mathworks, MA). Multi-channel ERP activity in 64 electrodes was created using Brainstorm to generate potential field maps from current source density profiles defined on the cortical surface. JICA was performed on the concatenated ERP/fMRI datasets using Matlab and the Fusion ICA Toolbox (http://mialab.mrn.org/software/fit/).

### Simulated brain activity

Three generative sources of activity, with different locations in the brain and different temporal profiles, were simulated to examine the effect of the spatial and temporal relationship between sources on jICA performance. FMRI activity of the sources was simulated as activation foci in a 3D whole brain MRI volume (64 × 64 × 33 voxels) in Talaraich space (Talaraich, 1988), using AFNI (Cox, 1996). Each source consisted of a cube (5 × 5 × 5 voxels, 4,000 μL) of active voxels with a homogeneous BOLD response. The magnitude of BOLD response for each source did not have detailed temporal structure but was scaled across representations of the data to simulate condition-dependent changes based on the experimental design (Figures 1C,D).

ERP activity of the sources was defined spatially on the cortical surface of the Colin brain (Holmes et al., 1998), as implemented in Brainstorm (Brainstorm 3.1), (Tadel et al., 2011). The cortical surface consisted of a mesh of 15,000 vertices with sample electric dipoles positioned at each vertex and oriented perpendicular to the cortical surface. Active vertices for each source were determined by projecting the volumetric fMRI activation foci to the cortical surface using Freesurfer (v 5.0), (Dale et al., 1999; Fischl et al., 1999). For each source, the spatial distribution of volumetric activity projected to the cortical surface was assigned a 1,000 ms time-varying current density profile selected from three forms found empirically (uniphasic or biphasic peak, and no response). The current density profiles for sources in right temporoparietal and left motor cortex were simulated as a gaussian-weighted biphasic sinusoid peaking at 100 ms and a uniphasic sinusoid peaking at 800 ms, respectively (Figure 2A, on the right). The prefrontal source was simulated as visible with fMRI but with no ERP response (i.e., it was uncoupled). Multi-channel ERP waveforms were simulated at 62 scalp electrode locations by forward projecting the current density profiles to the scalp surface of the Colin brain, using a 3-shell sphere head model, and the template 62 electrode locations of the 10–20 system, in Brainstorm.

### ERP—fMRI relationship

The relationship between the amplitudes of ERP and fMRI activity was simulated as either linear or non-linear to determine whether the jICA linear model could reliably recover non-linear signal relationships. For simulations with a linear ERP/fMRI relationship, the variation in the magnitude of fMRI activity across independent representations of the data was proportional to the variation in peak amplitude of the ERP (fMRI_mag_ = b^*^ERP_peak_). Simulations with a non-linear ERP/fMRI relationship where characterized by a saturating exponential across representations, [fMRI_mag_ = 1−exp(b^*^ERP_peak_)]. In all cases, the frontal source was simulated as an ERP-blind source such that ERP_peak_ = 0.

### Parametric and non-parametric experimental design

In separate simulations, the change in fMRI and ERP amplitudes across representations was designed to be correlated or uncorrelated, in order to characterize the impact of this latent variable on jICA performance. The change in fMRI and ERP amplitudes across representations was defined by the experimental design: In a parametric experimental design, the physiological activity varied parametrically across representations of the data, reflecting a systematic relationship with the experimental condition (for example, the stimulus level or task difficulty). In a non-parametric experimental design, the physiological activity varied non-systematically, reflecting a random relationship with the experimental condition (for example, if each representation of the joint ERP/fMRI data is a different unrelated experimental condition). In the parametric experimental design simulated here, the fMRI and ERP signal amplitudes for the three sources varied in coordination, resulting in a correlation between sources, effectively simulating a single functional brain network. In the non-parametric experimental design, changes in fMRI and ERP signal amplitudes were uncorrelated across representations of the data and between sources. Thus, ERP/fMRI sources were functionally independent, each presenting with a different random pattern of activation, effectively simulating three separate brain networks. A graphical illustration of the differences between the parametric and non-parametric design is shown in Figures 1C,D.

### SNR and number of independent representations of the ERP/fMRI data

The ability of jICA to accurately retrieve the sources was examined as a function of the number of independent representations of the ERP/fMRI data (22 values spanning the range 4–360) and the imaging peak SNR (PSNR), (22 linked fMRI/EEG values spanning the range −2 to 16.6 dB for fMRI activity and −8.6 to 10.9 dB for ERP activity), defined as the ratio between the squared maximum signal amplitude and mean-square error of the noise. The range of PSNRs in each imaging modality was selected based on experimental data for a single trial (the lowest PSNR) and an average of 72 trials of the same experimental condition (the highest PSNR) (Mangalathu-Arumana et al., 2012). This range encompasses two principal types of experimental designs, one with a small number of independent representations and high PSNR per representation (achieved through averaging across multiple repetitions of each experimental condition), and one with a large number of independent representations and low PSNR per representation (achieved by considering each trial as an independent representation of the data). A total of 484 simulations were performed for each combination of fMRI-ERP coupling (linear or non-linear) and experimental design (parametric or non-parametric).

Noise was added to the ERP and fMRI activity to simulate the different PSNR levels. For ERP signals, noise was simulated at the electrode interface as a zero mean Gaussian process and added to the forward projected current source density profiles at each electrode. The variance of the noise was specified as the ERP signal amplitude in the post-stimulus window divided by the standard deviation of the prestimulus baseline. For fMRI signals, Gaussian noise was applied at each voxel. The variance of the noise was specified as the amplitude of the fMRI signal divided by the standard deviation of a zero-mean noise.

### JICA

The fusion of fMRI and ERP data was performed using Multi-run jICA (Figure 2C; Mangalathu-Arumana et al., 2012). In Multi-run jICA, the variation in ERP and fMRI activity is described across independent representations of the data (e.g., experimental levels) in a single subject, as opposed to across subjects. In each simulation, jICA was used to extract jointly varying (across experimental levels) ERP and fMRI signal components that maximized independence in space and time.

Prior to jICA, fMRI, and ERP data sets were concatenated to create a joint-matrix, where each row consisted of the vectorized fMRI volume and ERP temporal profile across electrodes for a single observation. PCA was applied to the joint-matrix to whiten the signal and reduce the signal subspace to 20 components (for simulations containing more than 20 representations). JICA was then performed on the PCA-extracted components using the infomax algorithm (Bell and Sejnowski, 1995). Each joint component consisted of a spatial jICA-fMRI map and a spatiotemporal jICA-ERP map, containing linear projections of the fMRI and ERP signals covarying across representations of the data that maximized the statistical independence between jICA components.

jICA-ERP components were submitted to distributed source reconstruction to facilitate spatial comparisons with the corresponding jICA-fMRI activity and the generative source(s). Source localization of the jICA-ERP maps was performed in Brainstorm (http://neuroimage.usc.edu/brainstorm) using the weighted minimum norm estimate (wMNE) to solve the inverse problem for a distributed representation of electric dipoles located at each vertex on the cortical surface model and oriented perpendicular to the cortical surface (Brainstorm 3.0, Matlab, 2010b). The Colin head volume conductor and cortical surface models used to forward project the generative sources were used to reconstruct the cortical source activity from each jICA-ERP component.

### Statistical analysis

JICA-fMRI maps were amplitude thresholded at *p* < 0.01 relative to the normal distribution of activity across all voxels and jICA-fMRI components. A corrected map-wise cluster threshold of α < 0.05 was applied to identify regions of significant activity; defined relative to the chance distribution of cluster sizes across jICA-fMRI components, computed by spatial randomization of the voxel-wise activity within each component. JICA-ERP source maps were amplitude thresholded at *p* < 0.01 relative to the normal distribution of the post-stimulus activity across all vertices and jICA-ERP components.

### Source detection

The extent to which the jth ERP/fMRI source was extracted into the nth jICA component was characterized using the equivalence (spatial for fMRI, temporal for ERP) between the activity within the jICA component and the generative “true” sources. For fMRI, the relative contribution of the nth jICA-fMRI component to the activity of the generative source was defined as:

(1) $$\begin{array}{r} s_{jn}^{fMRI}=\frac{\bar{a_{jn}}}{\sum_{n=1}^{N}|\bar{a_{jn}}|} \end{array}$$

where (*a_jn_*) is the average activity across voxels in the true source volume, and *N* is the number of jICA components.

For ERP, the contribution of the nth jICA-ERP component to the jth source was defined using temporal correlation of activity across the source normalized components,

(2) $$\begin{array}{r} S_{jn}^{ERP}=\frac{\overset{M}{\underset{m=1}{\sum }}|r_{jnm}|}{\overset{N}{\underset{n=1}{\sum }}\overset{M}{\underset{m=1}{\sum }}|r_{jnm}|} \end{array}$$

where *r*_*jnm*_ denotes the correlation for the mth vertex of the true source. For the uncoupled prefrontal source, with no generative ERP model, *s*^*ERP*^ was evaluated using the ERP profile from the spatially nearest (temporal) source.

### Recovering the relationship between ERP and fMRI activity using jICA

In jICA of ERP and fMRI activity, the samples in each imaging modality are constrained by the same mixing matrix and are therefore correlated across representations of the ERP/fMRI data, reflecting the theoretical assumption that the fMRI and ERP activity are driven by a common neural source. In practice, ERP and fMRI activity from a common neural source may not be fully correlated. Such “uncoupling” may be due to differences in the sensitivity of each imaging modality to the location and temporal course of activity, and/or to non-linearities in neurovascular coupling (Zhang et al., 2008). When uncoupling occurs, fMRI and ERP measures from a neural source may be separated into more than one joint component; however, the components retain the spatial and/or temporal properties inherent to the common neural source.

The spatiotemporal overlap between components associated with a common neural source can be used to link them and recover the relationship between the neuroimaging measures. For each source, the relationship between ERP and fMRI activity can be recovered from the weighted mixing coefficients of each imaging modality (***A***_***ERP***_, ***A***_***fMRI***_),

$$\begin{array}{l} {\text{A}}_{ERP}={\text{DAQS}}_{ERP}\\ {\text{A}}_{fMRI}={\text{DAQS}}_{fMRI} \end{array}$$

where ***D*** is the pseudo-inverse of the *q* × *n* whitening matrix used to reduce the model order from *q* representations of the data to *n* components (n ≤ q), ***A*** is the *n* × *n* mixing matrix estimated during jICA, ***S***_***ERP***_ is a *n* × *l* matrix containing the ERP portion of the source matrix defined for *l* samples (electrodes x time) across n components, ***S***_***fMRI***_ is a *n* × *p* matrix containing the fMRI portion of the source matrix defined for *p* samples (voxels) across n components, and ***Q*** is a *n* × *n* sparse matrix with unit values along the diagonal specifying linked jICA components for the neural source being reconstructed. For the within-subject version of jICA used here, this process returns the relationship between ERP and fMRI signals across representations for neural sources that cannot be represented by the implicit linear relationship between fMRI and ERP within a single jICA component.

## Results

We examined the effect on within-subject jICA performance of several variables relevant to the fusion of ERP and fMRI (imaging SNR and number of independent representations of the data) as a function of the type of sources (parametrically, non-parametrically varying) and the relationship between changes in ERP and fMRI signal amplitude (linear, non-linear). The first set of simulations examined source recovery in the context of a parametric design and a linear (section Linear ERP-fMRI Relationship and Parametric Data Structure) or non-linear (section Non-linear ERP-fMRI Relationship and Parametric Experimental Design) relationship between ERP and fMRI signal amplitudes. The second set of simulations examined source recovery in the context of a non-parametric design (section Linear ERP-fMRI Relationship and Non-parametric Experimental Design).

### Linear ERP-fMRI relationship and parametric data structure

The first set of simulations examined jICA source recovery in the context of a parametric experimental design as described in sections Parametric and Non-parametric Experimental Design to SNR, and Number of Independent Representations of the ERP/fMRI Data. For these simulations, a systematic relationship existed between the experimental condition and the instantiations of the joint ERP/fMRI activity of all sources, resulting in a high functional correlation between the sources.

### Effect of SNR and number of independent representations of the data on source recovery

**Figure 3:**
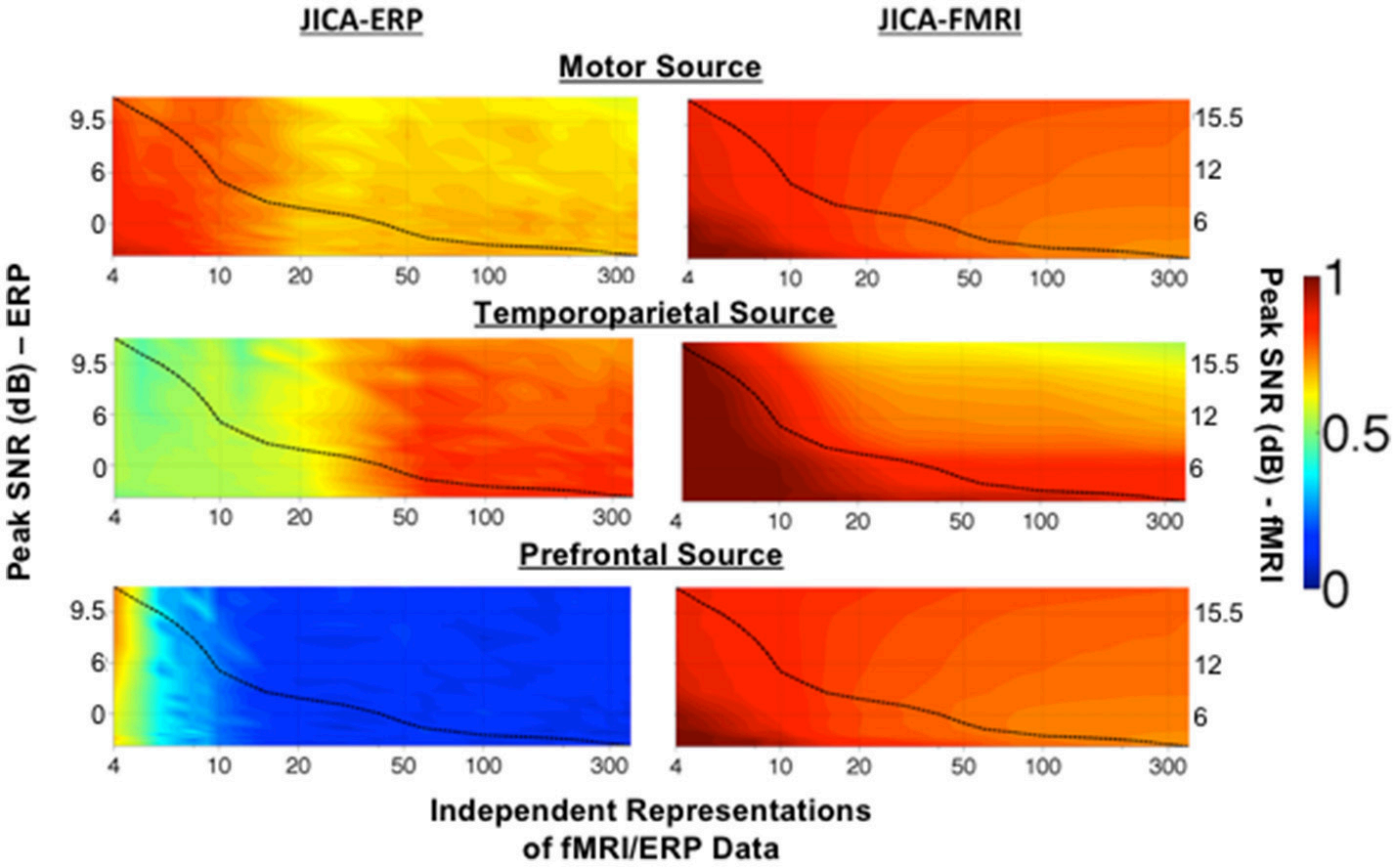
Maximum source detection values for within-subject jICA in a parametric experimental design. The degree of recovery by a single component of fMRI and ERP activity associated with each of the three sources, measured as the maximum fMRI and ERP source detection values across jICA components, [max_n_(*s*^*fMRI*^) and max_n_(*s*^*ERP*^) respectively], is shown as a function of peak SNR and number of independent representations of the ERP/fMRI data. The motor (uniphasic) and temporoparietal (biphasic) sources were simulated with linear coupling between the ERP and fMRI activity. The uncoupled prefrontal source consisted of an fMRI response only. Source detection values range 0–1, with a value of 1 corresponding to extraction of the complete source activity into a single joint component. A value of 0.5 indicates that 50% of the source activity was extracted into one component and the remaining activity was attributed to at least one additional component. A value of 0 indicates a finding of no activity associated with the source. The dashed line in each graph illustrates the √N relationship between SNR and number of independent representations of the data (e.g., trials, trial averages, etc.) in a typical experimental design (Mangalathu-Arumana et al., 2012). In an experimental design with a fixed number of trials, ERP/fMRI activity characterized by high peak SNR, may reflect trial averaging across a few experimental conditions while ERP/fMRI activity characterized by low peak SNR may reflect activity across many individual trials.

Figure 3 shows the degree to which each simulated source was extracted into a single component, as a function of the imaging PSNR and number of independent representations of the joint ERP/fMRI data. The degree of extraction was calculated using the ERP and fMRI source detection metrics, and could vary between 1 (signifying that all the source activity measured with ERP or fMRI was assigned to a single joint component) and 0 (signifying that no source activity was measured). A value of 0.5 indicates that half of the source activity was assigned to one component and the other half to one (or more) other component/s. Note that this analysis does not inform on whether the ERP and fMRI activity from each source was assigned to the same (joint) component or to different components.

The dashed line in each graph represents the √N relationship between SNR and number of independent representations of the data (e.g., trials, trial averages, etc.) in a typical neuroimaging experiment with a total of 360 measurements of ERP/fMRI activity (Mangalathu-Arumana et al., 2012), as a reference. In a typical experiment with a limited number of imaging measurements, there is a trade-off between PSNR and number of independent representations of the data, whereby a few experimental conditions (i.e., small number of representations) may be measured at high PSNR (averaging across trials) or many experimental conditions (i.e., large number of representations) may be measured at low PSNR (averaging across few trials for each condition, or no averaging as in a trial-wise experimental design).

The representation of ERP activity within a single jICA component (Figure 3, left), max*_n_*(*s^ERP^*), ranged 0.7–0.9 for the motor source (with uniphasic ERP), 0.5–0.9 for the temporoparietal source (with biphasic ERP), and 0.1–0.6 for the prefrontal source (with uncoupled ERP). The recovery of the motor source by a single component systematically decreased as the number of ERP/fMRI representations increased. The temporoparietal source showed a reverse trend, and was increasingly extracted into a single component as the number of ERP/fMRI representations increased. The prefrontal source had no ERP response associated with it. When the number of representations was small, residual ERP activity from the spatially nearest (temporoparietal) source was associated with the prefrontal fMRI source, however, the correlation was low (*r*^2^ ≤ 0.2).

**Figure 4:**
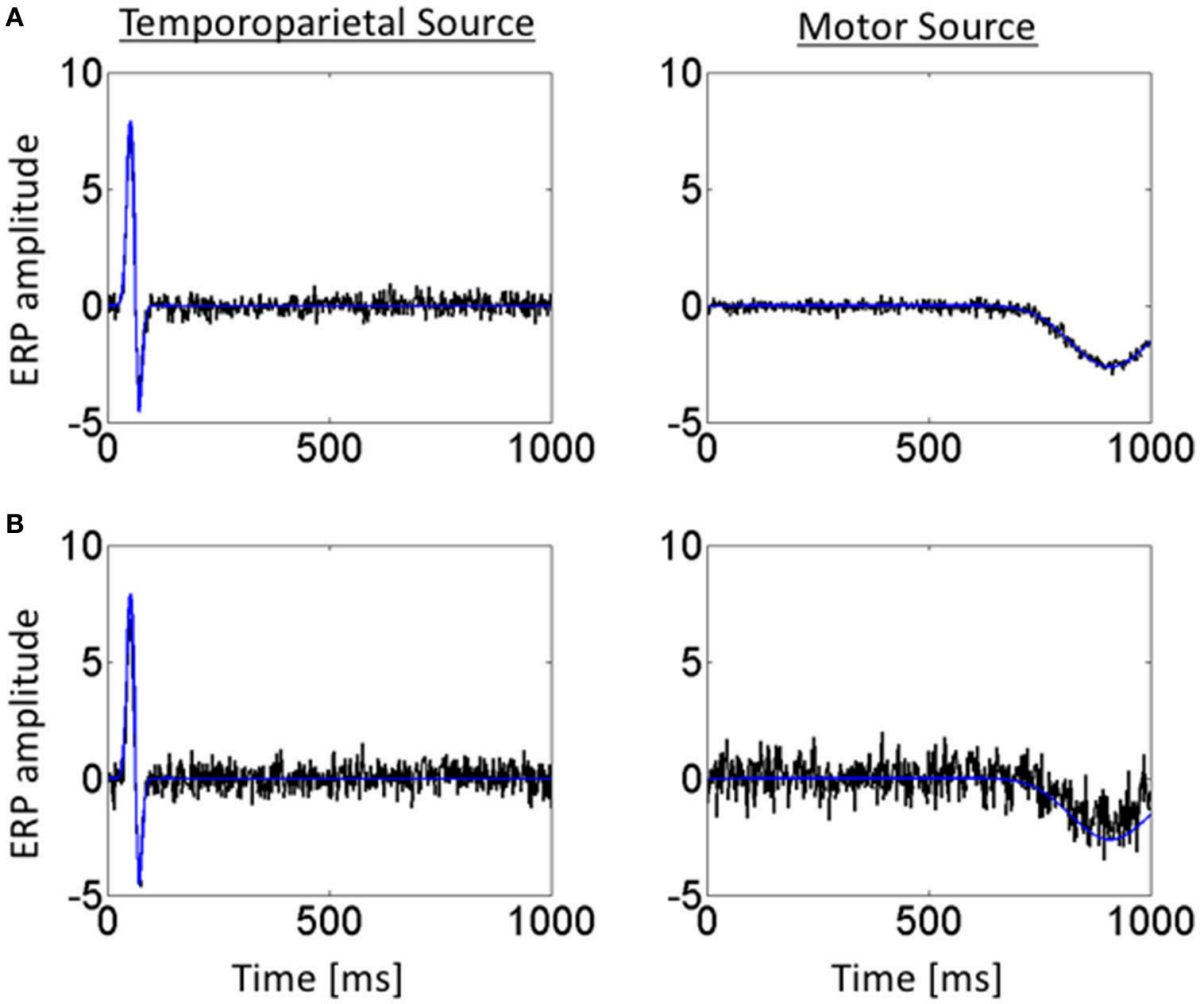
Temporal profiles of simulated ERP and retrieved jICA-ERP responses for representative vertices of the temporoparietal (left) and motor (right) sources. Temporal profiles are shown for the jICA-ERP source density (black line) and the simulated ERP source density profile (blue line; shown here without noise added for visual clarity) for, **(A)** high SNR (=10.9 dB) and a small number of independent ERP/fMRI representations (e.g., 6 experimental conditions) and **(B)** low SNR (=−8.6 dB) and a high number of independent ERP/fMRI representations (360 trials).

Figure 4 shows the temporal profiles of the jICA-ERP activity associated with the temporoparietal and motor sources (black) overlaid on the temporal profiles of the respective simulated sources (blue); at high SNR (=3.5) with the independent representations of the ERP/fMRI data corresponding to 6 experimental conditions (Figure 4A), and at low SNR (= 0.37) with ERP/fMRI representations corresponding to 360 trials (Figure 4B). The temporal profiles of the original and reconstructed sources were highly correlated (*r*^2^ > 0.98) in both cases, indicating accurate extraction of the sources with jICA.

The recovery of fMRI activity within a single jICA component (Figure 3, right), max*_n_*(*s^fMRI^*), ranged 0.8–1 for the motor source, 0.5–1 for the temporoparietal source and 0.8–1 for the prefrontal source. In each case, the recovery of the fMRI source into a single component increased with imaging SNR and decreased with the number of neuroimaging representations.

### Effect of imaging SNR and number of independent representations of the data on source separation

**Figure 5:**
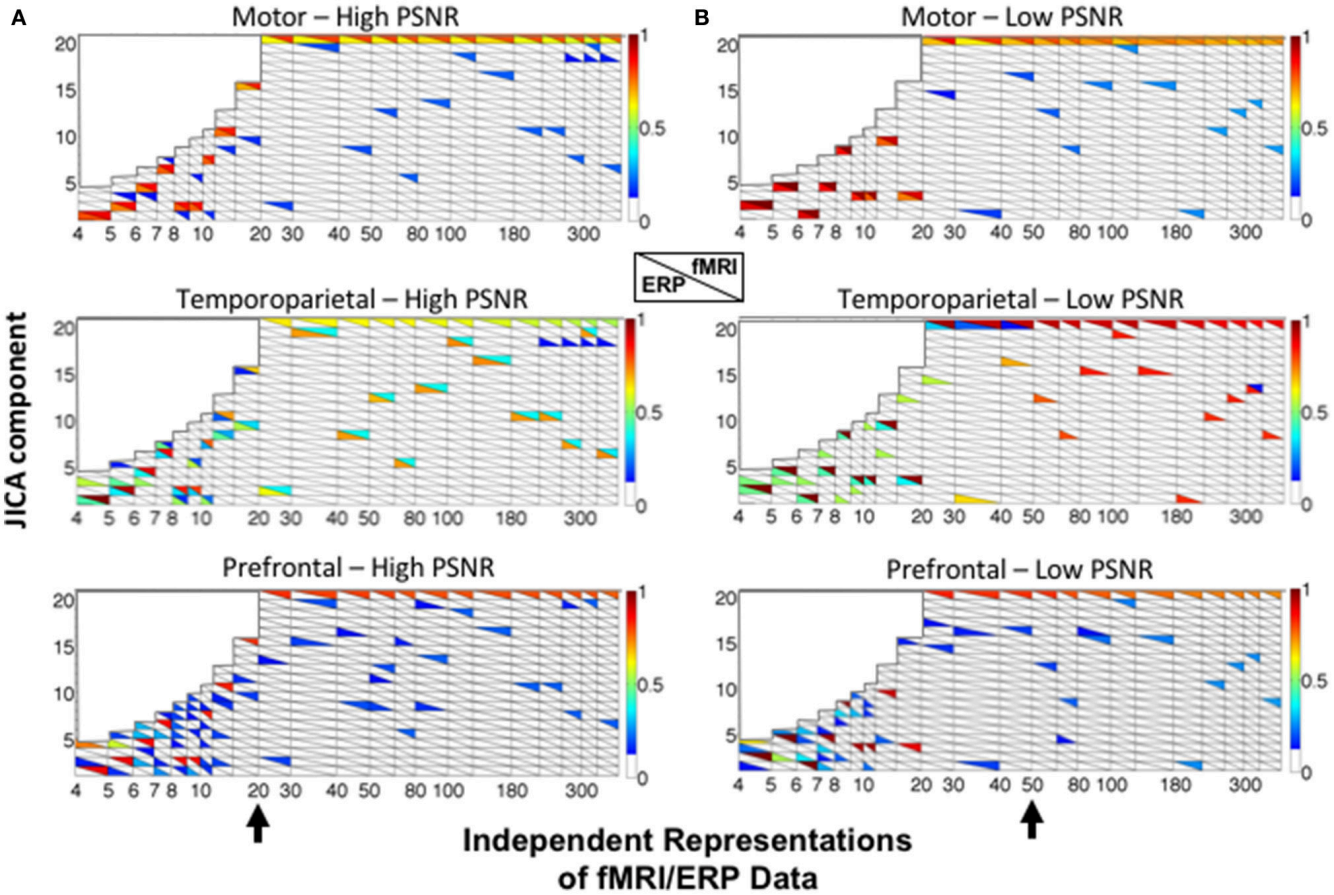
JICA source segregation with a linear relationship between fMRI and ERP activity in a parametric experimental design. JICA source detection values for ERP and fMRI (lower and upper triangles, respectively) of the motor (top panels), temporoparietal (middle panels) and prefrontal (lower panels) sources are shown for **(A)** high (ERP = 10.9; fMRI = 16.6 dB) and **(B)** low (ERP =−8.6; fMRI= −2 dB) PNSR. For the prefrontal source, ERP source detection values were calculated with respect to the spatially nearest (temporoparietal) source. The black arrow (bottom panels) indicates the number of independent representations of the ERP/fMRI data beyond which motor and temporoparietal ERP sources were separated into different components. The color bar denotes the source detection values of the original sources recovered in each component (*p* < 0.05 threshold).

Figure 5 shows the recovery of the motor (upper panels), temporoparietal (middle panels), and prefrontal (lower panels) sources across all 20 jICA components, as a function of the number of ERP/fMRI representations for high (A) and low (B) SNR. For each source, the ERP and fMRI activity recovered in each component is characterized by the ERP (*s*^*ERP*^; lower triangles) and fMRI (*s*^*fMRI*^; upper triangles) source detection values. Source detection values exceeding a threshold of 0.1 *(p* < 0.05) are shown, for clarity. This depiction shows the extent to which, ERP and fMRI signals associated with each source were captured in the same (max *s*^*ERP*^ and *s*^*fMRI*^ present in the same rectangle) or different (max *s*^*ERP*^ and *s*^*fMRI*^ in upper and lower portions of different rectangles) joint components.

The simulation results show that the fMRI activity associated with all three sources is recovered in the same joint component, irrespective of the SNR and number of independent representations of the ERP/fMRI data (component 20—upper right triangles). This finding is consistent with our previous empirical jICA results showing that fMRI activity associated with different neural sources of the P300 were carried by a single component in a parametric experimental paradigm (Mangalathu-Arumana et al., 2012). In contrast, the ERP activity associated with the motor and temporoparietal sources was consistently recovered into separate components. It is worth noting that while ICA does not inherently order components, sources that also account for a majority of the signal variance (such as the common fMRI source) will be consistently extracted (and ordered) during PCA dimension reduction (representations >20).

For the motor source (with uniphasic ERP), ERP and fMRI responses were recovered in the same component (colored squares) at both low and high SNR. A different trend was observed for the temporoparietal source (with biphasic ERP), wherein ERP and fMRI activity was increasingly recovered into different components as the number of independent representations increased (Figure 5—the point of component separation is indicated by a black arrow). At high SNR, residual fMRI activity was associated with the ERP component after the point of separation. For example, with 20 representations at high SNR, component 2 contained ERP activity and residual fMRI activity, and component 20 contained the primary fMRI activity. At low SNR, residual activity linking the fMRI and ERP components was observed for up to 50 representations of the ERP/fMRI data.

For the prefrontal source (with no ERP), ERP source detection was characterized relative to the spatially nearest (temporoparietal) source and was consistently low (<0.2). In no cases were the fMRI activity of the true prefrontal source and ERP activity of the temporoparietal source recovered into the same jICA component.

### Non-linear ERP-fMRI relationship and parametric experimental design

In this set of simulations, the relationship between changes in ERP and fMRI signal amplitudes was linear for the motor source and non-linear [1-exp(x)] for the temporoparietal source. The prefrontal source remained uncoupled as before. Changes in the ERP and fMRI activity of the three sources varied systematically across representations (trials, trial averages, etc.), consistent with a parametric experimental design, resulting in a high functional correlation between the sources.

**Figure 6:**
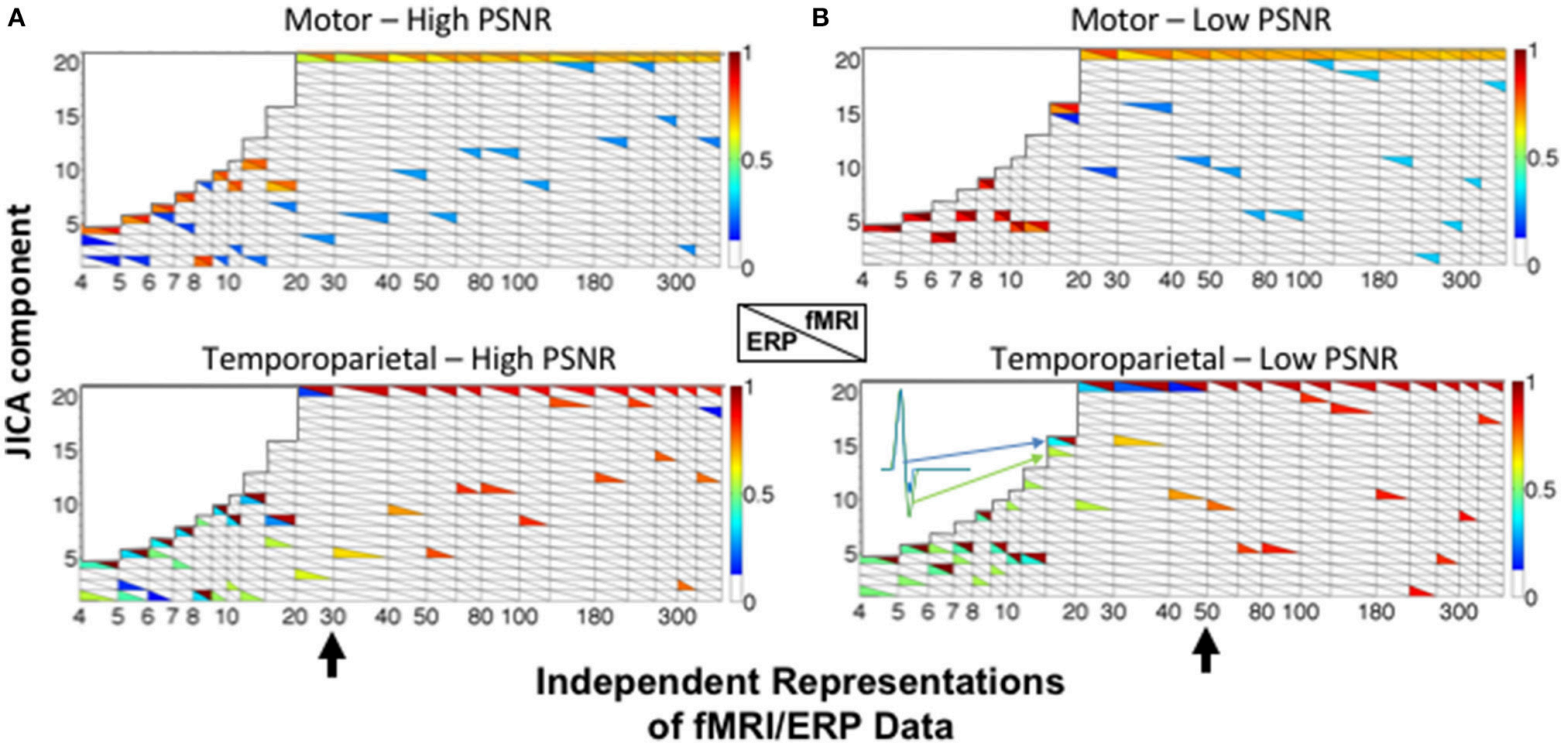
JICA source segregation with a non-linear (exponential) relationship between fMRI and ERP activity in a parametric experimental design. JICA source detection values for ERP and fMRI activity (lower and upper triangles, respectively) corresponding to the motor (linear relationship—top panels) and temporoparietal (non-linear relationship—lower panels) sources, at **(A)** high (ERP = 10.9; fMRI = 16.6 dB) and **(B)** low (ERP = −8.6; fMRI = −2 dB) PSNR. The inset illustrates the temporal profile of the biphasic source when it is split between a primary ERP component and a linked residual in the fMRI component. Other labeling conventions are the same as in Figure 5.

JICA performance was largely similar when the relationship between changes in fMRI and ERP signal amplitudes was non-linear. For the temporoparietal (biphasic) source, ERP and fMRI activity was recovered in different components (Figure 6). For small and moderate numbers of ERP/fMRI representations (<30 for high SNR; <50 for low SNR), the joint component containing the temporoparietal fMRI activity also contained significant residual ERP activity (*p* < 0.05), effectively linking the two components. For large numbers of ERP/fMRI representations (>50), the fMRI and ERP activity was recovered into different components. In contrast, ERP and fMRI activity associated with the motor (uniphasic) source was extracted into the same joint component regardless of the number of representations. FMRI activity associated with the temporoparietal source was also present in the joint component representing the motor source, reflecting the co-variation of sources across representations that is inherent to the parametric data structure and typical of most experimental designs.

### Linear ERP-fMRI relationship and non-parametric experimental design

In a final set of simulations, the impact of a non-parametric experimental design on jICA performance was examined. In this case, ERP-fMRI signal amplitudes were linearly coupled but varied randomly across independent representations of the data and between sources.

Similar to the results for the parametric experimental design, source detection with linearly covarying fMRI and ERP signals increased with SNR and number of independent representations of the data. Source detection values for fMRI ranged 0.4–1 (Supplementary Figure 1, right) for the three sources. However, for the non-parametric experimental design, the recovery of fMRI activity into a single joint component was dependent on SNR and the number of representations. Extraction into a single component was less likely at low SNRs and for small numbers of representations, (max*_n_*(*s^fMRI^*) = [0.4, 0.6]), and increased quickly along both dimensions (max*_n_*(*s^fMRI^*) = [0.8, 1]).

Similar to the parametric experimental design, jICA source detection for ERPs ranged 0.7–0.9 for the temporoparietal and motor sources, and 0.1–0.3 for the prefrontal (uncoupled) source (Supplementary Figure 1, left). Recovery of the temporoparietal (biphasic) ERP activity into a single joint component increased with SNR and number of representations of the ERP/fMRI data. Source detection of the motor (uniphasic) ERP activity decreased with the number of representations, indicating a splitting of the ERP activity across multiple components.

**Figure 7:**
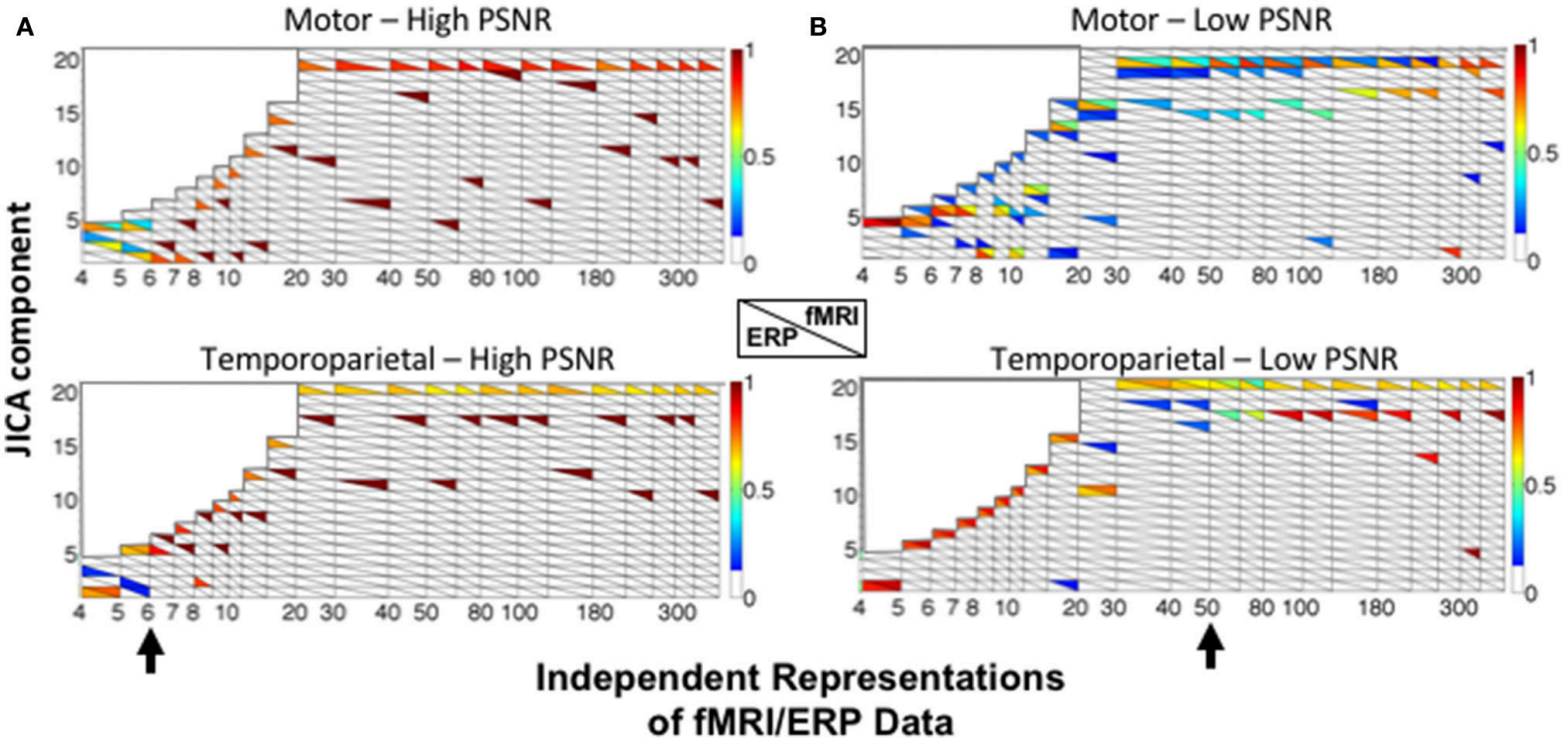
JICA source segregation with a linear relationship between fMRI and ERP sources in a non-parametric experimental design. JICA source detection values for ERP and fMRI activity (lower and upper triangles, respectively) corresponding to the motor (top panels) and temporoparietal (lower panels) sources, at **(A)** high (ERP = 10.9; fMRI = 16.6 dB) and **(B)** low (ERP = −8.6; fMRI = −2 dB) PNSR. Other labeling conventions are the same as in Figure 5.

Figure 7 shows the sources recovered across all jICA components. At high SNR (Figure 7A), the ERP and fMRI activity for each source was recovered into the same joint component for fewer than six independent representations of the data (e.g., experimental conditions). For larger numbers of representations, fMRI and ERP activity was recovered into separate components, with no overlap. At low SNR (Figure 7B), temporoparietal ERP and fMRI activity was recovered into a single component for fewer than 15 representations. As the number of representations increased, primary fMRI activity was recovered into a separate component, with residual fMRI activity linked to the ERP response for up to 80 representations of the ERP/fMRI data. Similar trends were observed at low SNR for the motor source.

### Recovering the relationship between ERP and fMRI activity using jICA

**Figure 8:**
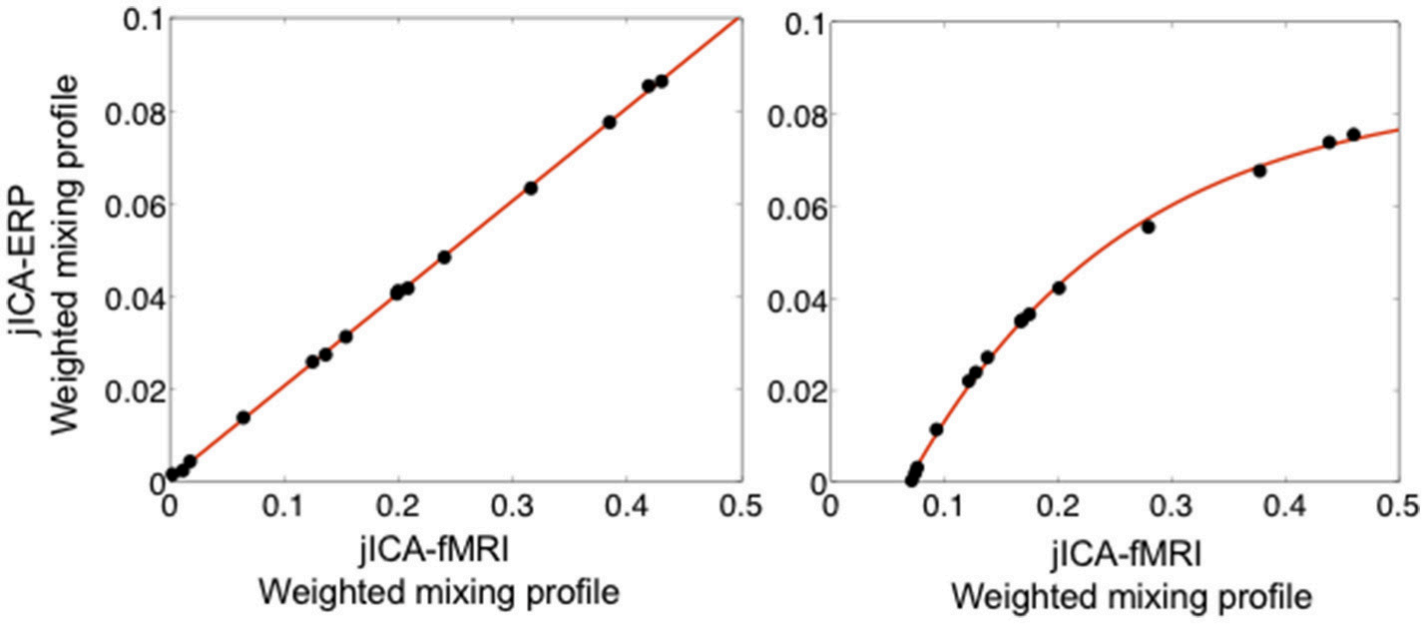
Relationship between fMRI and ERP activity of the temporoparietal source extracted using the weighted sum of jICA mixing coefficients, when the relationship was simulated as linear (left) and non-linear (exponential—right). The relationship is shown following jICA of a parametric experimental paradigm with 15 representations of the ERP/fMRI data (PNSR; ERP = −3 dB, fMRI = 5.7 dB). The data points represent the fMRI and ERP relationship for all components containing significant activity associated with the source (*p* < 0.05). The original simulated coupling relationship is denoted by the solid line.

Figure 8 shows the linear (left panel) and non-linear (right panel) relationships between ERP and fMRI activity recovered from the weighted jICA mixing coefficients of the temporoparietal (biphasic) source with 15 representations of the ERP/fMRI data. In both cases, the ERP and fMRI activity was split across two or more components, where one component contained primary activity from one modality (e.g., ERP) and residual activity from the other modality (e.g., fMRI). Both the linear and non-linear relationships between ERP and fMRI signals were accurately recovered. The result suggests that the jICA mixing profiles, weighted by their component activity, can be used to link multi-modal activity correlated along overlapping dimensions, e.g., in space and time for ERP, and in space for fMRI.

## Discussion

We used computer simulations to examine the performance of jICA as a function of imaging SNR, number of independent representations of the ERP/fMRI data (e.g., trials, trial averages, etc.), the ERP temporal profile (uniphasic, biphasic), and the relationship between the ERP and fMRI signals (linear, non-linear, uncoupled), for parametric and non-parametric experimental designs. JICA performance was evaluated based on: (1) The ability to retrieve the spatial and temporal attributes of each neural source in a single joint ERP/fMRI component (or to link separate ERP and fMRI components reflecting the same neural source); and (2) The ability to retrieve activity associated with spatiotemporally independent neural sources into separate components. The simulations demonstrate that the recovery of ERP and fMRI activity from a common neural source into a single joint component depends on SNR, number of representations of the neuroimaging data, and the type of experimental design. The results provide quantitative estimates of jICA's performance in recovering neuroimaging activity across these dimensions, that can be used to guide ERP/fMRI experimental design and data fusion analyses.

When neural sources are correlated across representations of the ERP/fMRI data, as in a parametric experimental design, the simulations showed that fMRI activity was consistently retrieved into a single jICA component, reflecting a functionally defined brain network. Whether ERP activity was retrieved into the same (single) component as the fMRI activity depended on the source temporal profile: for the motor source (with a uniphasic ERP profile) it was, but for the temporoparietal source (with a biphasic ERP temporal profile) activity was recovered in a separate component that contained residual fMRI activity from the same source. This finding is consistent with prior experimental results (Mangalathu-Arumana et al., 2012), and favors the interpretation that jICA applied within-subject recovers functional networks irrespective of differences in the temporal patterns of activity within the brain areas that define the network. Put simply, each combination of linked jICA components together defines the spatiotemporal profile of a functionally independent brain network. When neural sources vary independently across representations, as in a non-parametric experimental design, the recovery of fMRI activity into a separate component for each source increases with imaging SNR and the number of representations of the ERP/fMRI data. This outcome is expected for jICA, and ICA approaches more generally, since in this scenario the sources reflect distinct functional networks whose spatial (and temporal) patterns of activity are uncorrelated.

The finding, observed in both the parametric and non-parametric experimental designs, that ERP and fMRI activity were recovered into different jICA components as SNR and the number of neuroimaging representations increased is consistent with simulations examining the effect of information diversity and order selection on the outcome of data-driven fusion analyses (Adali et al., 2015b). In neuroimage fusion analyses, over-parsing the data due to improper order selection can manifest as decoupling between neuroimaging measures originating from a common neural source (e.g., Figure 7 for large numbers of representations). This effect is also impacted by SNR, dependencies between representations and the relative diversity of the multimodal signals themselves. ERP, compared to fMRI, activity is generally more variable across representations of the data (in both temporal and spatial dimensions) and therefore contributes more prominently to the separation of independent sources with jICA. When the model order is incorrect, differences in the spatiotemporal patterns of ERP and fMRI activity increasingly dominate the source separation as SNR and the number of representations of the data increase.

The simulations show that JICA performance is more robust to incorrect estimates of model order at low SNR and for dependencies between representations of the data (i.e., task levels in a parametric experimental designs). In these conditions, the residual ERP activity is maintained within the fMRI component associated with the same neural source. These residual interactions between components can be used both to recover common neural sources when model order is incorrect and, when combined with the jICA mixing coefficients, to recover non-linear relationships between ERP and fMRI activity.

A limitation of jICA for multimodal neuroimage fusion lies in the presumption of a common mixing profile between neuroimaging measures. For the within subject ERP/fMRI fusion analysis presented here, this constraint implies that the fMRI and ERP activity associated with a source covaries across independent representations of the data (i.e., experimental conditions). However, under certain circumstances, differences in the origin (neural vs. hemodynamic) and spatiotemporal resolution of the ERP and fMRI signals can lead to non-linear and uncoupling relationships. In such cases, jICA can recover the underlying neural sources and can estimate the non-linear relationship using the weighted sum of jICA mixing coefficients across linked components. In spite of this, it will be important for future studies to compare within subject jICA with other data-driven approaches, including canonical correlation analysis (CCA), parallel ICA, and independent vector analysis (IVA), to identify the conditions in which each is most appropriate for multimodal analysis of neuroimaging data within subject (Correa et al., 2008, 2010; Calhoun and Adali, 2009; Adali et al., 2015b). For example, by relaxing the constraint of a common mixing profile, these approaches can directly estimate non-linear and uncoupling relationships between imaging modalities and may be more sensitive to variations across brain networks. In contrast, for ERP/fMRI analysis within subject, where the number of independent representations of the data is small and the association between imaging modalities is mostly linear, jICA may provide a more reliable estimate of the underlying brain networks (Adali et al., 2015b).

In sum, the simulations presented here provide a framework for understanding the impact of experimental design on jICA of multimodal neuroimaging data. When jICA is applied within-subject, the separation of independent brain sources is maximized in a non-parametric experimental design, but is more susceptible to a loss of correspondence between ERP and fMRI signals at high SNR when model order is over-estimated. The best performance, maximizing both cross-modal data fusion and the recovery of neural sources into components that define functional brain networks, occurs for a moderate number of independent representations of the ERP/fMRI data (~10–30), as in a mixed block/event related experimental design. Importantly, the type of relationship between ERP and fMRI activity, whether linear, non-linear or uncoupled, does not in itself impact jICA performance. Data-driven approaches are particularly advantageous when the relationship between ERP and fMRI activity is unknown, or when it is suspected to be non-linear or uncoupled due to an underlying pathology. In jICA, the type of relationship between ERP and fMRI activity is accurately represented in the common profiles (i.e., mixing coefficients). When applied within-subject, jICA provides an unbiased approach to characterize the relationship between ERP and fMRI activity across brain regions.

## Author contributions

JM-A, SB, and EL: conceived and designed the simulations; JM-A: implemented the simulations, performed the analysis, and drafted the initial manuscript; All authors interpreted the results and critically reviewed the manuscript. SB and EL finalized the manuscript.

### Conflict of interest statement

The authors declare that the research was conducted in the absence of any commercial or financial relationships that could be construed as a potential conflict of interest. The reviewer JS and handling Editor declared their shared affiliation, and the handling Editor states that the process nevertheless met the standards of a fair and objective review.

## Supplementary material

Supplementary Figure 1 Maximum source detection values for within-subject jICA in a non-parametric experimental paradigm. JICA source separation of the fMRI and ERP activity, computed as the maximum fMRI and ERP source detection values across jICA components, [max_*n*_(*s*^*fMRI*^) and max_*n*_(*s*^*ERP*^) respectively], is shown for the three sources as a function of the peak SNR and number of independent representations of the ERP/fMRI data. The motor and temporoparietal sources were simulated with linear coupling between the ERP and fMRI activity. The prefrontal source, consisting of an fMRI response only, was uncoupled. Other labeling conventions are the same as in Figure 3.
