# Supplementary figures and images for: Optimizing Within-Subject Experimental Designs for jICA of Multi-Channel ERP and fMRI
Source: Front Neurosci. 2018 Jan 23;12:13. doi: 10.3389/fnins.2018.00013 (PMC5787094; doi:10.3389/fnins.2018.00013)

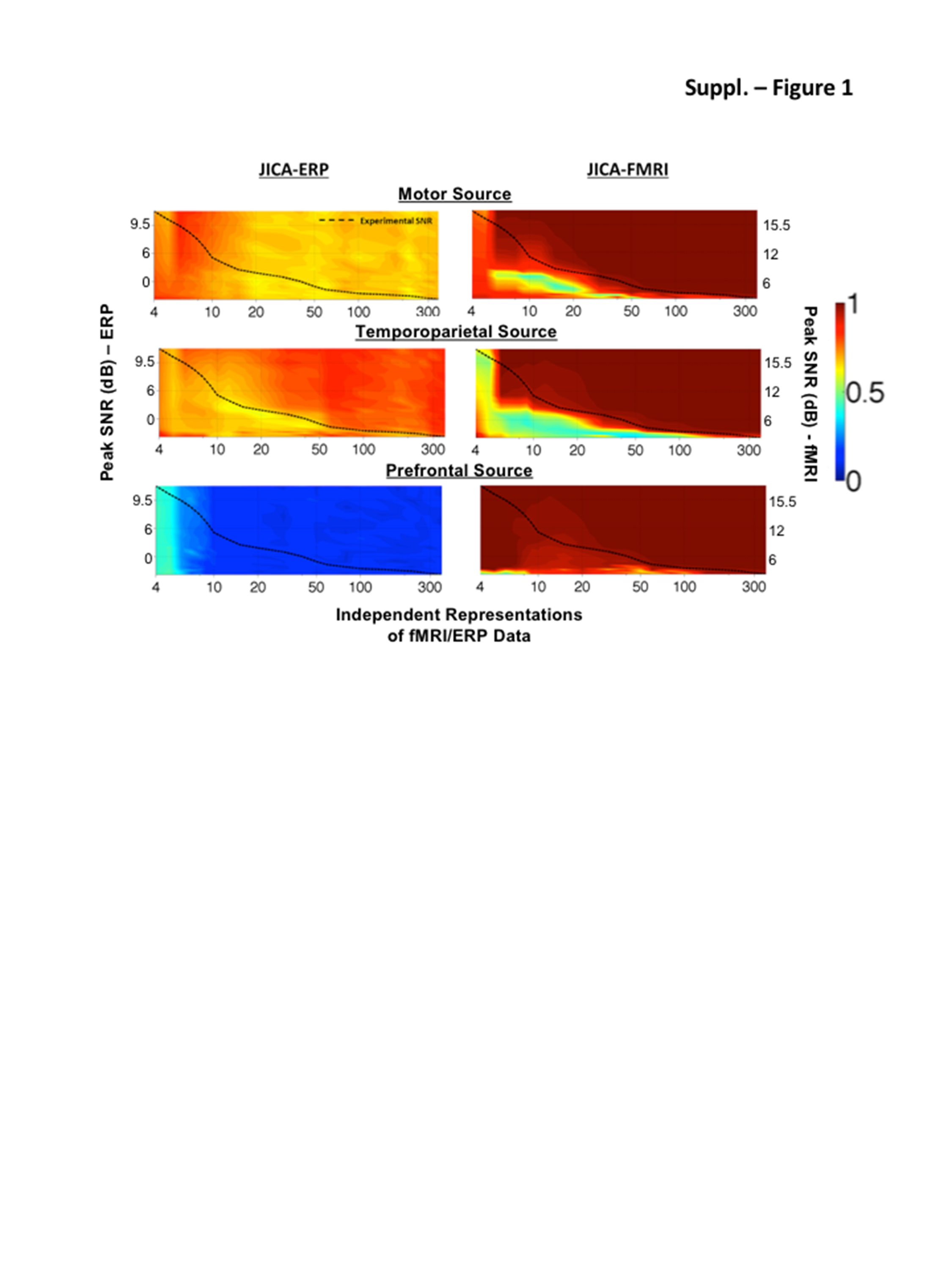

Supplement: Supplementary Figure 1 — Maximum source detection values for within-subject jICA in a non-parametric experimental paradigm. JICA source separation of the fMRI and ERP activity, computed as the maximum fMRI and ERP source detection values across jICA components, [maxn(sfMRI) and maxn(sERP) respectively], is shown for the three sources as a function of the peak SNR and number of independent representations of the ERP/fMRI data. The motor and temporoparietal sources were simulated with linear coupling between the ERP and fMRI activity. The prefrontal source, consisting of an fMRI response only, was uncoupled. Other labeling conventions are the same as in Figure 3. [file Image1.TIFF]
